# Toward a Logic of the Organism: A Process Philosophical Consideration

**DOI:** 10.3390/e24010066

**Published:** 2021-12-30

**Authors:** Spyridon A. Koutroufinis

**Affiliations:** Institute for Philosophy and History of Science, Technology, and Literature, Technical University of Berlin, Strasse des 17. Juni 135, 10623 Berlin, Germany; spyridon.koutroufinis@campus.tu-berlin.de

**Keywords:** theory of organism, systems biology, biological modeling, theory of dynamical systems, differential equations, control parameters, process ontology, Biological Statistical Mechanics

## Abstract

Mathematical models applied in contemporary theoretical and systems biology are based on some implicit ontological assumptions about the nature of organisms. This article aims to show that real organisms reveal a logic of internal causality transcending the tacit logic of biological modeling. Systems biology has focused on models consisting of static systems of differential equations operating with fixed control parameters that are measured or fitted to experimental data. However, the structure of real organisms is a highly dynamic process, the internal causality of which can only be captured by continuously changing systems of equations. In addition, in real physiological settings kinetic parameters can vary by orders of magnitude, i.e., organisms vary the value of internal quantities that in models are represented by fixed control parameters. Both the plasticity of organisms and the state dependence of kinetic parameters adds indeterminacy to the picture and asks for a new statistical perspective. This requirement could be met by the arising Biological Statistical Mechanics project, which promises to do more justice to the nature of real organisms than contemporary modeling. This article concludes that Biological Statistical Mechanics allows for a wider range of organismic ontologies than does the tacitly followed ontology of contemporary theoretical and systems biology, which are implicitly and explicitly based on systems theory.

## 1. Introduction: On the Concept of the Organism

The term ‘organism’ first appears in biological texts in the late 17th century [1] (p. 777). Since the 19th century, it has represented a central concept of biology that together with the term ‘life’ defines the biological field of study. After the discovery of the structure of the DNA in the 1950s, 20th century biology became extremely focused on the concept of the gene. Searching for particular genes that supposedly determine particular features of the organism or cause specific diseases is much easier than establishing a holistic view of the organism. This attitude, however, is reminiscent of the man who is searching under a lamppost for a key that he has lost on the other side of the street. In the wake of gene-centered biology, the formation of the organismic phenotype was reduced to a preformed ‘translation’ of a genetic blueprint to a complex material structure. In accordance with this view, the evolution of species was interpreted as a sequence of processes of natural selection on accidentally occurring mutations of genes. Under the combined pressure of neo-Darwinian evolutionary theory and gene-centered molecular biology, the concept of organism fell into oblivion and was almost excluded from biological reasoning. In the last decades, nonetheless, this situation is radically changing. In the 21st century the concept of organism is regaining its primary role in biological thought. To a large extent, this is due to two seminal developments: Firstly, the deep crisis in the modern understanding of genes and, secondly, the increasing awareness of all organisms’ ability to restructure their genetic and even physiological constitution if needed. As this elementary ability of all organisms demonstrates that they can autonomously manipulate their genetic equipment, it is obvious that neither the dynamics nor the autonomy of an organism can be explained by referring to its genome. There are good reasons to expect that 21st century biology will be the result of the conviction that the explanation of biological phenomena requires a sophisticated theory of the organism. However, the emergence of such a theory first requires the elaboration of a logic of the organismic mode of being that proceeds from detailed descriptions of the phenomenon of organism.

The main aim of this article is to outline abstract rules of a *logic of the organism* that demonstrates both the *autonomy* of organismic self-organization and the principal inability of systems-theoretical reduction of organisms to do justice to this autonomy. This is not to be understood as an attempt to develop any specific logical formalism. The logic of the organism aims at an abstract description of essential, but almost always implicit thought principles, on which causal explanations of organismic phenomena should be based. All explanations of said phenomena are rooted in an understanding of matter, energy, causality, information, and other scientific terms that always imply a certain philosophical ontology.

In Section 2 of this article, the common essence of organismic phenomena is sought. This author suggests that it makes sense to distinguish between three increasingly abstract levels of analysis of organismic phenomenality as it is presented in the literature. Then follows the analysis of different types of causal-ontological explanations of the phenomenon of organism. Its main focus is on the system-ontological foundations of contemporary systems-theoretical explanations of organismic phenomena as well as on the process ontology of Alfred N. Whitehead. Departing from a critical consideration of mechanistic explanations within modern systems biology, which operate with differential equations, a ‘logic of systems biological mechanisms’ is formulated. This logic can be seen as the abstract core of the system-ontological consideration of the organismic phenomenon. The ‘logic of systems biological mechanisms’ was developed from the study of a large number of articles published by systems biologists and philosophers of science representing the so-called ‘New Mechanical Philosophy’ or ‘New Mechanism.’ Said logic consists of two abstract rules, which, although never explicitly mentioned, imply the most essential common element of the mathematical mechanisms of the models employed by systems biologists, although these rules are never explicitly mentioned.

In Section 3 an attempt is made to formulate rules of a ‘logic of the organism,’ which gives the organismic autonomy an abstract and concise expression. It is shown that this logic prepares the ground for a particular understanding of selfhood, resonates with Jakob von Uexhüll’s concept of ‘Umwelt,’ and requires a radical re-conception of the idea of the state space, which biology has adopted from physics. It is also demonstrated that this logic is consistent with the core ideas of Whitehead’s process ontology. Particular attention is paid to the essential and irreconcilable differences between the ‘logic of the organism’ and the ‘logic of systems biological mechanisms.’ The third section does not aim to question the practical use of the systems-theoretical reduction of organisms for biomedical research. What is criticized here is the tacit ontological assumption that organisms are nothing but dynamic self-organized material entities, the causality of which can in principle be described by systems biological mechanisms.

Section 4 is dedicated to the new project of Biological Statistical Mechanics. It is asserted that while its proponents use systems biological mechanisms to a large extent, they do not fall into the wide error of endowing them with ontological relevance. This is due to the fact that they are aware of the essential and therefore irreducible indeterminacy of all kinds of biological entities, which forbids their strict mathematical description in the form of differential equations.

Section 5 is the conclusion. One of its central messages is that the project of Biological Statistical Mechanics harmonizes well with the two abstract rules of the logic of the organism and that its philosophical foundation can therefore be supported by the process ontological understanding of the organism.

Finally, it should be noted that this author does not claim to have formulated a mature logic of the organism. Such an endeavor would require the incorporation of the biophilosophies of Aristotle, Henri Bergson, George Canguilhem, Jakob von Uexküll, Hans Jonas, Kurt Goldstein, Viktor von Weizsäcker, Gilbert Simondon, Raymond Ruyer, and other thinkers. These authors can be seen as main protagonists of a discipline that could be described as ‘classical biophilosophy.’ Unfortunately, not only are their works ignored by almost all theoretical and systems biologists, but they are also almost absent from the publications of mainstream Anglo-Saxon philosophers of biology. After the beginning of the decline of the gene-centered leveling of the concept of organism, both modern biology and the Anglo-Saxon philosophy of biology are increasingly dominated by different variants of systems-theoretical thinking. This can be seen very clearly in the formalisms of systems biology. In view of this situation, the author is convinced that any attempt to introduce a mature logic of the organism on the basis of classical biophilosophy must begin with a critical examination of both systems-theoretical reductionism and the works of those philosophers of science, and in particular of biology, who follow the ‘New Mechanical Philosophy.’ The present study is therefore to be understood as the first step toward establishing a logic of the organism. It aims to show the limits of systems-theoretical reductionism of the present in relation to this logic.

## 2. Phenomenal and Ontological Analysis of the Organism

Philosophers may consider the concept of organism through inherently different methodical perspectives. In this article a distinction between two essentially different philosophical methods for approaching this concept is suggested: The term ‘phenomenal analysis of organism’ subsumes approaches to the nature of the organism that are limited to the most complete possible description of the phenomena essential for living beings. In contrast, what is referred to in this article as the ‘causal-ontological analysis of the organism’ aims at the theoretical justification of the phenomenal analysis. It aims at the exposition of the organismic phenomenality as a logical consequence of the relations between entities or processes, the understanding of which requires the usage of fundamental hypotheses concerning the nature of matter, energy, information, causality, time, and other basal scientific and philosophical concepts. Those hypotheses may comply with the implicit ontological assumptions of mainstream biosciences, physics, and philosophy of science but can also question them and introduce new ways of metaphysical thinking [2,3]. The following are examples of causal-ontological explanations of the phenomenon of the organism: Aristotle’s explanation of the structure and behavior of the organism through the concept of the soul; Descartes’ explanation of morphogenesis and metabolism by mechanical forces; Claude Bernard’s Physiology; the old- and neo-vitalistic explanations, e.g., by Georg E. Stahl and Hans Driesch; the molecular biological reduction of the organism to the genetic information and the genetic program; the systems-theoretical explanation of organisms, mainly founded by Ludwig von Bertalanffy, which through its extension by the theory of non-linear dynamic systems has become the mainstay of current theoretical and systems biology; and Whitehead’s process ontological approach to the organism.

However, it is not always clear which of the two methods an approach uses. Some approaches combine both types of analysis. A good example of this is the theory of autopoiesis. Its founders Humberto Maturana and Francisco Varela developed a new terminology and language to describe the organismic phenomenon, but in their writings they often propose a systems-theoretical reduction of this phenomenon, which is typical of a causal-ontological analysis. Furthermore, there are biophilosophical approaches to the concept of the organism that cannot be assigned to either of the two categories. Both Immanuel Kant’s theory of the organism in his *Critique of Judgment* and Jakob von Uexküll’s theoretical biology are the best examples of those approaches. Kant’s reduction of all experiences and causal events to the structure of pure reason does not allow us to consider biological phenomena as facts that exist independently of consciousness. This must necessarily deprive any biophilosophy based on Kant’s transcendental philosophy of any ontological relevance. Based on Kant’s transcendental idealism, von Uexküll suggests an epistemological foundation not only of the concept of organism, but of biology as a whole. While Kant’s influence is clear in the first chapters of his main work *Theoretical Biology*, in the later chapters his thinking emancipates itself from Kant’s purely epistemological method [4].

### 2.1. Three Levels of Phenomenal Analysis of Organism

This article distinguishes between three successive levels of phenomenal analysis, in which the phenomenon is treated on a higher level of abstraction than on the previous level.

#### 2.1.1. Organism as the Body of a Living Being

The term ‘organism’ is a scientific term that denotes the same entities as the pre-scientific term ‘living being.’ According to phenomenological philosophy, the experience of our ‘lifeworld’ (a term derived from the German term ‘Lebenswelt’) gives us an immediate, non-discursive knowledge of what living beings are. Our lifeworld experience (lebensweltliche Erfahrung) of living beings precedes the discursive talk of organisms and thus forms the logical basis of the latter. This asymmetrical relationship of dependency is reflected very clearly in the phrase ‘organism of a living being’ [5] (p. 214), while the expression ‘living being of an organism’ is never uttered. From the view of this phenomenological position, the term ‘organism’ refers to the bodies of living beings. On this basis, the organism can be defined as follows: “The term organism describes the system of structures and processes by which a living being is able to live and carry out its characteristic activities” [5] (p. 214, translated by S.K.).

However, this approach to the concept of the organism presupposes that we have a prescientific, non-discursive ability to recognize which of the beings that inhabit our environment are living beings. According to phenomenologically oriented philosophers, we would not be able to recognize living beings as such if we did not have a living body of our own, i.e., if we did not live ourselves. We can only therefore speak abstractly about organisms and establish biological discourses because we are *embodied* living beings [6] (p. 163). Without the direct experience of our embodied existence within our lifeworld we would not be able to distinguish living beings from complex nexuses of interrelated physicochemical events that (the nexuses) are spatiotemporally coherent but inanimate dynamical systems. Hans Jonas summarizes this line of thought in his famous proposition “life can be known only by life” [7] (p. 91). Phenomenology traces the scientific concept of organism back to “its cognitive source, which is the lived experience of our bodily being”, in other words, “empathy is a precondition of our comprehension of the vital order, in particular of the organism as a sense-making being inhabiting an environment”—“[e]mpathy in this sense encompasses the coupling of our human lived bodies with the bodies of other beings we recognize as living, whether these be human, animal, or even [...] bacteria” [6] (p. 163).

The phenomenological argumentation also provides a philosophical justification for a tradition of approaching the essence of the living being that has existed since antiquity—the listing of the properties common to all animated beings. Such a list could contain the following properties, for example: organization (hierarchical groups of cell organelles within cells, of cells within organs etc.), metabolism, reciprocal interdependencies in organic functions, growth, development, agency and autonomy, self, homeostasis, processing of information, irritability and semiosis, the ability to distinguish self from non-self, reproduction (even in non-reproducing organisms cells are reproduced), adaptability, evolvability [8] (p. 222f.), [9], and selectability (organisms are often considered the most efficient level of selection [1] (p. 803)). This list of properties can be supplemented, which is why such lists are heavily dependent on the respective author. What is important here, however, is that we can only therefore cognize these properties because we observe them in beings that we have previously empathically recognized as living beings. Consequently, the direct experience of our embodied existence enables us to recognize not only living things, but also their essential properties, by means of which we can try to define life scientifically.

The phenomenological approach has been criticized for the fact that the sense of sight, on which it is based, reveals the spatiotemporal borders of biological individuals only in a few special cases: “The problem is that phenomenal individuation does not work as soon as one considers living things other than higher vertebrates. Common sense cannot say where the individual is when the focus is on siphonophores, aspens, fungi, or slime mold—to take but few examples […]. Moreover, a cell, for instance, nicely fulfills all our criteria of individuality, raising the important question of whether a multicellular organism is better seen as one individual or a community of (cellular) individuals” [10] (p. 249). This criticism must be taken seriously because the idea of biological individuality is essential for understanding the concept of organism. Nevertheless, from a phenomenological point of view, this criticism can be countered as follows: We know intuitively what it means to be alive, even if we cannot always recognize whether a being is an organismic individual or not. The idea of life does not coincide with the ideas of the biological individual and the organism, but includes them. Since we live ourselves, we have an intuitive knowledge of what life is that enables us to recognize living wholes, even if we cannot always judge whether they are individuals or are compositions of these. Our empathic knowledge allows us to recognize siphonophores, aspens, mushrooms, slime molds, and multicellular organisms as beings that are related to us in an absolutely elementary way—as living. Otherwise we would not have recognized them as the appropriate category of embodied beings for which complex questions such as biological individuality can be explored.

Empathic knowledge, however, is not a discursive, scientific one. If it were, there would be no need to write articles like this one. Aspects of a theory of the organism that cannot be dealt with from a phenomenological point of view make it necessary to introduce two further, non-phenomenologically oriented levels of the phenomenal analysis of the organism. Nevertheless, the phenomenological access to the organism precedes them logically and temporally.

#### 2.1.2. Emphasis on Metabolism as the Most Essential Feature of Organisms

The understanding of the organism as a living being’s body emphasizes one side of the living entity: its material constitution. However, “[t]he other side, the dynamical, is its unity as a nexus of processes that reciprocally depend on each other” [1] (p. 805, translated by S.K.). The physicochemical processes inside the living being generate its material parts and during morphogenesis (e.g., embryogenesis or metamorphosis of insects) transform them so that “new bodies (of the same living being) can be generated”, which, due to their continual transformations, undergo modifications that give rise to new forms of causally interpenetrated processes [1] (p. 805, translated by S.K.), which in turn generate new bodily structures. The dialectic between the permanent transformation of the body and the persistence of the nexus of causal interdependent processes that constitutes the organism is captured by the idea that “living beings are a category *sui generis*”—the category of *continuants* [11] (p. 126). In metaphysics, the term ‘continuants’ denotes individuals which persist even though they are subject to permanent changes in space and time [11] (p. 126), [1] (p. 806). However, two things should be noted: First, in contrast to inorganic beings, an organism does not suffer its permanent spatiotemporal changes passively, but rather causes and controls them actively. Second, the persistence of an organism does not result automatically, but is an achievement of its special processuality, which is why it can be lost—organisms are precarious beings. Organisms are continuants because they actively resist their decay and repeatedly create the conditions for their continued existence.

The change of focus from the living being’s material constitution to its self-continuation through its own processuality allows us to separate the concept of organism from the (permanently altering) living body and to elevate it to the principle that pertains to a living being’s continuous material transformation and governs it. Thus conceived, the term ‘organism’ becomes a principle of identity that refers to the self-continuation of an altering nexus of processes and not to materiality. As Hans Jonas suggested, the organism can be understood as a process: “the living organism exists as a constant exchange of its own constituents, and has its permanence and identity only in the continuity of this process, not in any persistence of its material parts. This process indeed is its life, and in the last resort organic existence means, not to be a definite body composed of definite parts, but to be such a continuity of process with an identity sustained above and through the flux of components” [12] (p. 47). Jonas’ consideration of metabolism as the most fundamental activity of an animated being is summarized in the following definition: The organism is a nexus of reciprocally conditioning processes that maintains or purposefully varies its material form above and through the continuous exchange of its material constituents.

By emphasizing the exchange of matter, the second level of phenomenal analysis elevates metabolism to the most essential feature of the organism. Nevertheless, the question arises whether inanimate self-organized systems that exchange matter with their environment can also be assigned a form of metabolism. Is the following statement true, which draws a sharp line between organisms and inanimate systems? “Continuity of the organization of the body *precisely through* constant change of material—that is the form of persistence, through which living beings differ from all inanimate things” [11] (p. 141, translated by S.K.). In contemporary physics and chemistry, the energetic and material openness of inorganic systems is considered a necessary condition for self-organization. So called ‘dissipative structures,’ such as whirlpools, flames, and the Belousov-Zhabotinski reaction (B.Z. reaction), display spontaneously generated complex dynamics. The BZ reaction generates a spatiotemporal pattern of molecular concentration, which has often been compared to biological pattern formation or morphogenesis. Nonetheless, those self-organized behaviors emerge only after the underlying systems are exposed to border conditions that are extrinsic to them, namely to external gradients of matter (and/or energy), forcing them to import and export matter (and/or energy) from and to their environment respectively. However, the passive dependence of inorganic self-organized systems on conditions imposed on them externally stays in a sharp contrast to the essential ability of any living being to autonomously generate nearly all conditions that the maintenance and steering of its metabolism require, as we will see in the exposition of the third level of phenomenal analysis. Often, this most fundamental ability of all intact organisms is attributed to their capacity to process genetic and other forms of molecular information and, in the case of animals, neuronal information, which inorganic systems cannot. But this widespread opinion lacks a reliable theoretical basis, since the use of the concept of information in biology is problematic. All forms of biological information have semantic aspects, the understanding of which is necessarily tied to philosophical concepts such as purpose, teleology, meaning, and value. However, there is currently no information theory that adequately incorporates these terms, so that information theory cannot bridge this gap between organisms and inorganic self-organized systems.

Understanding metabolism also requires addressing another central question in contemporary theory of the organism: Do the spatial boundaries of an organism coincide with its morphological ones? The answer to this question, which raises the issue of what exactly belongs to the body of an organism, is essential for a deeper understanding of the phenomenon of metabolism, as this occurs between the spatial boundaries of the organism and its environment. This question is not trivial because, according to some biologists, the morphological boundaries of some organisms do not coincide with their functional ones. This distinction between the two types of boundaries is based on two different understandings of organisms: First, the organism is a *morphologically* coherent unit that is separated from its environment by a continuous material boundary such as skin or membrane. Second, the organism is a *physiological-functional* unit of causally dependent objects that do not all belong to the same spatial continuum. Proponents of the physiological-functional definition of the organism argue with examples commonly associated with the idea of the ‘extended phenotype’: “In this way, a spider web is part of the spider’s organism, just like an odor trail laid by an ant should be counted among its organism, or how many structures built by organisms can be identified as ‘external organs.’ From a morphological point of view, the boundary between an organism and its environment is therefore not clear” [1] (p. 802). This applies even to processes that take place outside the body of the organism, but are central to its life. In coral reefs, for example, an important part of the physiology of calcification occurs outside of the coral body [1] (p. 802). These examples show that some objects that for the morphological consideration of the organism belong to its environment for the physiological-functional consideration must be regarded as parts of the organism: “The rabbit’s den is just as much part of its functional organism as the hair is part of its morphological organism” [1] (p. 803).

The undoubtedly central problem of the discrepancy between the (visible) morphological and (abstract) physiological-functional boundaries of some organisms cannot be dealt with in this article. Here we must be content with the observation that in most organisms their morphological unit coincides with their physiological-functional unit. The problem of the boundaries of the organism is directly related to the issue of the relationship between the organism and its environment. This question in turn has to do with the problem of the autonomy of the organism, since organisms, unlike inanimate objects, regulate their relationship to their environment autonomously. The incomparable autonomy of organisms is the subject of the third level of phenomenal analysis.

#### 2.1.3. Emphasis on the Autonomy of Organismic Self-Organization—The Need for the Formulation of a Logic of the Organism

After the discovery of the DNA, 20th century biology was re-grounded on the concept of the gene. In recent decades, however, the concept of the organism has moved ever closer to the center of biological thinking. As mentioned earlier, this development is also due to the increasing awareness of the ability of organisms to act on their genomes. Living cells and multicellular organisms have a huge number of ways to *reorganize* their DNA [13,14,15,16]. One of the leading geneticists, James A. Shapiro, speaks about the “natural genetic engineering” of organisms which means that “living cells can engineer their DNA”, so that “[t]he capacity of living organisms to alter their own heredity is undeniable” [13] (p. 2). At present, there is a rapidly growing number of publications in the fields of genetics, epigenetics, and developmental plasticity that demonstrate the ability of organisms not only to reorganize their own phenotype but also their genomes [13,14,15]. The following examples demonstrate the ability of organisms to radically transform their morphological, genetic, and physiological structure:

(i) There are many notable examples demonstrating the striking ontogenetic *plasticity* of multicellular organisms. The well-known theoretical biologist Mary Jane West-Eberhard is noted for arguing that phenotypic and developmental plasticity play a seminal role in animal evolution and speciation. In her influential book *Developmental Plasticity and Evolution,* she defines ‘plasticity’ or ‘phenotypic plasticity’ as “the ability of an organism to react to an environmental input with a change in form, state, movement or rate of activity” [17] (p. 34). This ability enables ontogenetic development to respond to challenges imposed by the conditions of life as well as to genetic challenges. West-Eberhard provides one of the most remarkable examples of animal plasticity: A goat, which was born without front legs, “adopted semi-upright posture and bipedal locomotion from the time of its birth” [17] (p. 51). When after a year of bipedal life the goat died due to an accident, it had developed behavioral specializations similar to those of kangaroos, and other bipedal mammals, such as “the ability to hop rapidly when disturbed” [17] (p. 51). The clinical autopsy revealed many morphological changes in its innervation, musculature, and skeleton, such as enlarged hind legs, a curved spine, an unusually large neck, a thorax similar to that found in kangaroos, and a wide sternum resembling that of an orangutan [17] (p. 52). According to evolutionary theorist and geneticist Eva Jablonka, those correlated phenotypic changes are due to flexible mechanical reorganization, new regulatory interactions, and “exploratory processes followed by stabilization at the cellular, physiological and behavioral level”, whereas at the molecular level, developmental plasticity “can involve epigenetic changes in regulatory systems” [16] (p. 101).
(ii) Experiments with larvae of the fruit fly *Drosophila melanogaster* demonstrate the same radical plasticity of developing individuals that enables them to appropriately react “to a challenge that the species can never before have encountered” [16] (p. 102): In artificially created lines of flies a gene that resists a specific drug was linked to various tissue-specific promoters, so that it could only be expressed in some tissues but not in others. Then the fly larvae were fed with food contaminated with a toxic concentration of the drug. The flies would only then survive if the gene were expressed in additional tissues. Indeed, after a developmental delay, the promoter activity had been extended over other tissues, so that the resistance gene was expressed in the gut and other organs, thus enabling the larvae to tolerate the otherwise lethal dose [18].
(iii) There are examples of organisms responding to different kinds of challenges through the reorganization of their material structure that cannot be subsumed under the technical term ‘plasticity’ because this term does not cover the entire spectrum of cases in which an organism transforms its structure. The highly influential French biophilosopher, epistemologist, and physician Georges Canguilhem describes an experiment providing evidence for what he refers to as the ‘vicariance of function’, meaning the ability of organs to serve multiple purposes: A placenta of a pregnant rabbit, that was extracted from the uterus and placed in the peritoneal cavity, grafted itself onto the intestine and nourished itself normally. When the graft was completed, the rabbit’s ovaries were operationally removed, which suppressed the function fulfilled by the corpus luteum during pregnancy. This resulted to the immediate abortion of all the placentas inside the uterus so that only the placenta that was grafted on the intestine survived and came to term. “Here is an example where the intestine behaves like a uterus, even, one could say, with more success than the uterus itself” [19] (p. 89).
(iv) Fascinating examples of genetic and epigenetic reorganization are encountered when, due to interspecific or intraspecific hybridization, “two, often very different, genomes” are present “in the same nucleus” [16] (p. 104f.). This gives rise to serious problems, such as the disruption of the usual pattern of gene expression and the improper formation of chromosome pairs during meiosis. The hybrids, however, sometimes succeed in overcoming these difficulties through “widespread, extremely rapid, reproducible, targeted, genetic and epigenetic modifications” [16] (p. 105).
(v) There is also striking evidence that both unicellular organisms and cells of multicellular organisms implement various modifications of their material structure which purposefully direct the course of intracellular molecular reactions [20] (pp. 2, 4), [21] (p. 35). In addition, as it will be shown in Section 3 of the present article, there is an impressive body of evidence that the shape modification of a cell plays a crucial role in governing metabolic and functional processes [22] (p. 5).

Other than the second level of phenomenal analysis of organisms, the third level does not underscore the centrality of metabolism for the understanding of organisms. Rather, it emphasizes the various fundamental organismic processes that enable the metabolism to continue even under extremely adverse conditions. In contrast to metabolism, which is a phenomenon familiar to the lay observer, those processes can only be revealed and addressed by scientific exploration. Thus, the present analysis requires a higher level of abstraction above the admittedly essential insight that organisms maintain or lawfully vary their material form despite the permanent and complete exchange of their material constituents.

All the examples described above demonstrate that cells and multicellular organisms can autonomously manipulate their morphology and physiology up to the molecular and even genetic level of the latter. This makes obvious that neither the dynamics of an organism nor the autonomy of its processuality can be reduced to its genome. In addition—and this is more essential—the intensity of the internally and autonomously performed reorganization of organisms down to the molecular structure of their body is a unique biological ability that is unparalleled in the inorganic realm. The reorganization that organisms perform at their behavioral, physiological, morphological, and molecular-genetic level enables them to radically restructure their body so that, even under extreme internal and external circumstances, they can generate most conditions required for the continuation of their metabolism. This highly fundamental biological faculty, which is ubiquitous in the realm of life, clearly transcends the abilities of all inorganic self-organizing systems: Organisms do not simply react to external gradients of matter and energy but *manipulate the inner conditions of the formation and maintenance of their morphological structure and their physiological and genetic dynamics* in a way that enables the continuation and, if necessary, the purposeful modification of their metabolism. The autonomous manipulation of the inner-organismic conditions, which determine the internal functions and external actions of the organisms, *identifies the distinctiveness of the organismic mode of being*. This ability often allows organisms to modify their environment in a way that serves the preservation and steering of their metabolism. This means, however, that the *autonomy* and *depth* of organismic self-organization cannot even approximately be attained by the most complex examples of physical or chemical self-organization of inanimate matter. Thus, there are good reasons to expect that 21st century biology will be erected around the insight that the explanation of biological phenomena demands a sound theory of the organism. In the opinion of this author, the arising of such a theory requires the elaboration of a *logic of organismic causality* that meets two conditions: First, it does justice to the radical autonomy of organismic processuality, which is evidenced by all the above mentioned examples. Second, it occupies a middle position between the phenomenal analysis and the causal-ontological analysis. In Section 3 of this paper, a detailed outline of what has been elsewhere labeled ‘logic of organisms’ [23] (p. 31f.) will be provided. Nonetheless, the *explanation* of organismic phenomenality can be achieved only by the causal-ontological analysis of the organism. This subject will be addressed immediately.

### 2.2. The Causal-Ontological Analysis of the Organism

Scientific explanations presuppose specific ontologies. They are implicitly and more rarely explicitly underlain by metaphysical assumptions about the nature and essential properties of entities, the interactions of which give rise to the phenomena requiring an explanation. Those philosophical assumptions are the most fundamental theoretical propositions that many if not all natural sciences of a particular historic era share. The history and present state of Western thought shows that both the biological sciences and their philosophical foundation have been grounded on three main ontological traditions succeeding one another.

#### 2.2.1. The Substance-Ontological Approach

For over two thousand years, between the era of Socrates and the birth of mechanistic physics in the 17th century, the metaphysics of *substance* was the backbone of occidental philosophy and science. The Greek term ‘ousia’ (οὐσία), initially meaning a person’s possession, was made by Plato and Aristotle the mainstay of classical Greek philosophy. Latin authors translated it later as ‘substance.’ Aristotle refined the concept of substance and assigned it a number of distinct meanings, one of the most influential of which became that of *essence*. The substance of a particular being, understood as its essence, is the atemporal, and thus eternal, reason of all its features, which must be attributed to it in order to distinguish it from other beings [24] (p. 106) (VII, 3, 1028b 34). Under the influence of Aristotelian substance ontology, the concept of essence became the main principle of reasoning about nature. It was considered to be the eternal active principle that shapes a being’s material constitution, while matter was reduced to an entirely passive receiver of action. Thus, according to Aristotle’s theory of causality, the essence of a particular being acts on its material constituents to some end, called ‘final cause’ or ‘telos.’ His worldview simply forbids considering a natural process, which is controlled merely by mechanical forces, as being able to achieve the kind of ordered result attained by an appropriately formed organic structure that serves the purpose of staying alive, like an organism or an organ, rather than degenerating into chaotic malformation [25] (p. 51–52) (II, 8, 198b 33–199a 28). In Aristotelian biology the timeless essence of an organism is considered a non-material causal factor that acts on matter and orders it. In his seminal work *De Anima* (*On the Soul*), Aristotle introduced two ideas that exerted a great influence on the history of biological thought: Firstly, that the essence of a “natural body having in it the capacity of life”, i.e., a body with biological organization, is its soul and, secondly, that all processes occurring in an organism are determined by its soul [26] (p. 8) (II, 1, 412a 19–21), [27]. The growth of an organism is not determined by its material structure but by its soul, particularly by the part of it that Aristotle calls the ‘nutritive soul’ [26] (p. 72) (III, 11, 434a 22–25). It is the essence or nutritive soul of a growing organism that determines its metabolism and directs growth toward a particular final state. In other words, in Aristotle’s biology the agent of growth (and nourishment), the nutritive soul, is not reducible to something physically present such as the material constellation of the growing organism. Thus, in sharp contrast to contemporary materialistic biosciences, an essential feature of Aristotelian biology is that the formation and maintenance of an organism are determined by an immaterial causal factor. The central role of essence in the explanation of organism formation and persistence makes it the primary common element in the biological thought of ancient, medieval, and early modern times.

#### 2.2.2. The System-Ontological Approach and the Logic of Systems Biological Mechanisms

The decline of Aristotelian ontology was due to the scientific revolution initiated by the introduction of the Copernican heliocentric system in the Renaissance. In the 17th century, philosophers and physicists replaced Aristotelian causality with mechanical contact-forces (Bacon, Galileo, Hobbes, Descartes) or action-at-a-distance-forces (Newton). Their ontology can be labeled as ‘systems ontology.’ From its beginnings in the 17th century to the present day, systems ontology has been anchored in an abstract understanding of the term *system*: A ‘system’ is a set of elements that are connected to one another through certain relationships—in Ludwig von Bertalanffy’s words, “A system can be defined as a set of elements standing in interrelations. This means that elements, p, stand in relations, R, so that the behavior of an element p in R is different of its behaviour in another relation, R′” [28] (p. 55). Systems ontology totally reverses the main principle of Aristotelian philosophy of nature by reducing essential features of any actual being to its material elements and their relations or interactions. Thus, for principal reasons, since its very origin, this ontology rejects any form of explanation of natural phenomena based on the concept of essence. However, systems ontology is not free from overlapping with substance ontology. It rather emerged after the ancient and medieval concept of substance was radically reconceived, especially in Descartes’ metaphysics [23] (pp. 23–24). In the 17th century, Newton succeeded in describing the dynamics of the solar system without using any Aristotelian form of explanation. Newtonian mechanics provided the example of mechanistic systems ontology par excellence. In the mid-18th century, based on Newtonian mechanics, Kant showed that not only the planetary orbits but even the solar system’s generation could be explained without recourse to any non-mechanical cause. The penetration of biological thought by this ontology began with Descartes’ attempt to explain the formation of the embryo and the sustainment of an organism in terms of mechanical contact forces between bits of matter in his work *Description du corps humaine* [29] (pp. 252–255). In the mid of the 19th century, the Germans Carl Ludwig, Hermann von Helmholtz, and Emil du Bois-Reymond, inspired by Descartes’ methodology, tried to base physiology on the assumption that the function of a whole organism can be explained by summing the results obtained from the analysis of the properties of isolated organs [30] (p. 259). In 1865 French physiologist Claude Bernard introduced a considerably more synthetic view of the organism, according to which living beings consist of diverse material processes that determine each other synergistically [30] (p. 272). Due to his emphasis on synergism, Bernard may be considered the first non-Cartesian materialistic physiologist. After a short revival of substance ontology in the neo-vitalistic biology of the early 20th century, the establishment of mathematical theoretical biology in the 1930s and especially the rise of molecular biology twenty years later facilitated the final breakthrough of materialistic interpretations of the organism. The result of this development is that almost all contemporary bioscientists and philosophers of biology subscribe to some version of materialism stemming from systems ontology.

In the last twenty years, due to the emergence of synthetic and systems biology, theorization of the organism came under the strong influence of mathematically formulated systems-theories. In the wake of that development—which was anticipated by pioneers of mathematical biology, such as Ludwig von Bertalanffy, Conrad Waddington, Stuart Kauffman, and Brian Goodwin [28,31,32,33,34,35]—organisms are considered complex dynamical systems of nonlinearly interacting biomolecules. Leading biologists today regard systems biology as supporting the renaissance of the concept of organism. This assessment reflects the fact that systems biology is antagonistic to gene-centered molecular biology in one important respect, as it supports a change in attention from DNA to a systems-theoretical consideration of organismic processes. One of the central tasks of systems biology is the study of complex cellular processes. Since a primary goal of this discipline is to contribute to a better understanding and medical treatment of diseases such as cancer and Parkinson’s, there is a close connection between systems biology and medical-pharmaceutical research and industry.

Influenced by the paradigm of complexity and self-organization, contemporary theoretical- and systems biologists consider organisms energetically and materially open self-organizing dynamic systems that operate far from thermodynamic equilibrium. A system is defined as a *dynamic system* if its state at any given moment can be described in terms of a limited set of time-dependent, or *state variables* x(t) = x_1_(t), x_2_(t), …, x_n_(t), for which a mathematical function F can be formulated that creates a lawful connection between states at times t and t + δt. The properties of this function reflect the causal relationships at work between the elements of the system. The set of state variables (x_1_(t), x_2_(t), ..., x_n_(t)) spans an abstract space, the so-called ‘state space’ of the system. The development of a dynamic system—which is usually represented by trajectories in the state space—is not only the result of the function F but depends also on a group of externally set control parameters. The following formula is an abstract depiction of a dynamic system:

x(t + δt) = F(x(t), p, δt); p = p_1_, p_2_, …, p_m_ [36] (p. 40)

The function F is the most central element of the model. In most cases F is a system of coupled nonlinear differential equations. In the last formula, the letter p represents a set of *control parameters*. During a process of calculating the variables, the control parameters are usually kept constant and their values are determined by the modelers.

It deserves special attention that in systems biology the values of the control parameters are determined by the modelers, as this reveals a crucial aspect of this discipline. In classical mathematical sciences, as they were conducted by Galileo, Newton, Leibniz, Boltzmann, Einstein, Schrödinger, Heisenberg, and others, first one had to have a certain hypothesis about a causal relation which, in the second step, would be translated into a mathematical formalism. In carrying this out, a single method was applied: dynamic systems were represented by systems of equations solved under certain control parameters, the values of which were dictated by the nature of the elements of the system under examination. In contrast, the criterion of quality of systems biological models is not whether they faithfully represent the real nature of organisms and biological processes but whether they facilitate the further medical and biological research by enabling predictions and formulating new hypotheses. Therefore, a large number of different methods can be used at the same time in systems biology if this helps to outline a formalism and the control parameters under which this formalism enables the desired predictions [37]. *Utility rather than theoretical stringency is the criterion for assessing adequacy in systems biology*. Thus, systems biology should be seen as being closer to engineering than to science. Therefore, systems biological models of organismic processes should be considered as models for predicting the behavior and not as models for explaining the real causal relations between the parts of an organism. Precisely this is reflected in the freedom of the modelers in determining control parameters, since the latter often represent the hypothetical strength of the interactions between the organismic elements, while these interactions at the same time strongly influence the course of the phenomena being modeled. Due to the essential role of control parameters in systems biological modeling, later in this section special attention is paid to the parameter determination by the modelers.

The practical meaning of their research often forces life scientists to find ways to manipulate the course of biological processes—any form of successful medical therapy is mainly a desirable artificial phenomenon. In sciences, real or hypothetical causal structures that produce or predict the course and outcome of natural or artificial phenomena are generally referred to as *mechanisms*. Leading advocates of what is often described as ‘New Mechanical Philosophy’ or ‘New Mechanism’ argue that in many fields of science what is considered a satisfactory description of both natural and artificially generated phenomena requires providing a mechanism able to predict the course of those phenomena. As “life scientists commonly seek to uncover the mechanism responsible for the phenomenon of interest” [38] (p. 322), in the life sciences the course of phenomena is described and predicted by genetic, metabolic, biochemical, and other kinds of mechanisms. Due to the commitment of systems biology to the practical service of the life sciences, much of its research can be understood as the discovery, description, and creation of mechanisms. In systems biology as well as in contemporary philosophy of science, systems of differential equations that are supposed to explain observed phenomena or predict new ones are often referred to as ‘mechanisms.’ Thus, in biological modeling the end-states of biological events are calculated by mathematical mechanisms.

In the last decades several definitions of the term ‘mechanism’ have been introduced in New Mechanical Philosophy and in philosophy of biology. According to Bechtel and Abrahamsen, “[a] mechanism is a structure performing a function in virtue of its component parts, component operations, and their organization. The orchestrated functioning of the mechanism is responsible for one or more phenomena” [39] (p. 423). For Glennan, “[a] mechanism underlying a behavior is a complex system which produces that behavior by the interaction of a number of parts according to direct causal laws”, where the expression “direct causal laws” means that there are no intermediate parts between the interacting parts [40] (p. 52). Recently, a new version of this definition, the so called ‘minimal mechanism,’ has been introduced by Glennan and Illari: “A mechanism for a phenomenon consists of entities (or parts) whose activities and interactions are organized so as to be responsible for the phenomenon” [41] (p. 92). All mechanisms are mechanisms *for* something [41] (p. 92). Machamer, Darden, and Craver (MDC) emphasize the role of “the activities in mechanisms” because “[a]ctivities are the producers of change” and “types of causes” [42] (pp. 4–6). They state, however, that entities and activities determine each other, for they are interdependent correlatives [42] (p. 6). According to MDC “[m]echanisms are entities and activities organized such that they are productive of regular changes from start or set-up to finish or termination conditions” [42] (p. 3). The “set-up conditions” consist of both the start conditions (relevant entities and initial conditions) and the enabling conditions (e.g., border conditions which are often labeled ‘constraints’) of a phenomenon. By “termination conditions” MDC mean a “privileged endpoint, such as rest, equilibrium” or an activated state [42] (p. 11f.). Together with the “intermediate entities and activities”, i.e., the entities and activities that produce the end state from the start [42] (p. 12), the set-up conditions constitute the Explanans while the termination conditions are the Explanandum or, in some cases, the prediction of a new not yet observed phenomenon [42] (p. 21).

In systems biology it is always a computer simulation that shows how the explanandum results from a mathematical model consisting usually of differential equations [43] (p. 985). The same applies to simulations, the aim of which is not to explain an observed phenomenon, but to predict a new phenomenon that would occur under specifically manipulated conditions. At present, computer simulations of both small and large systems of equations are considered mechanistic explanations [44] (p. 363). The dynamics of those systems cannot be foreseen from the parts of the proposed mechanism and their interactions because the mechanism’s structure contains (mostly interwoven) feedback loops, the dynamics of which can only be displayed by a computer simulation. Complex mathematical models consisting of coupled differential equations have been introduced among other things for the computation of the cell cycle [45], genetic and metabolic oscillations, signal pathways within and between cells, and the prediction of the development of spatial patterns during embryonic morphogenesis (e.g., [46,47]). Mathematics has become indispensable in contemporary biological explanations. Therefore, in systems biology mechanisms are always mathematical models. In order to understand how systems biologists operate with mechanisms in their research we must look at mathematical models. What we will find out about the general logic of systems biological mechanisms is valid no matter which of the possible definitions of mechanism that were introduced above underlies a particular mechanism.

In systems biology, depending on the problem to be solved, a variety of different methods can be employed [37] (p. 536). Systems biologists, who model organismic processes as systems of differential equations, often focus on the modeling of the dynamics of genetic, metabolic and signal pathways [48,49,50,51,52,53]. They also study the behavior of larger network systems constituted by coupling these pathways [45,54], such as might occur in embryogenesis. From the perspective of the theory of dynamic systems, the final-state-directedness of embryogenesis, cell cycles, and other final-state-directed phenomena are thereby reduced to the dynamics of an enormously complex system of positively and negatively coupled biomolecular reactions, represented by positive and negative feedback loops in the corresponding mechanisms.

In order to demonstrate how this approach works, an exemplary case of the mathematical analysis of a biological system, implemented with differential equations, is presented. The following model, published a few years ago, is a characteristic example of the way systems biologists work: A small network of biomolecular reactions between three interacting proteins of the frog’s egg is described by three coupled differential equations [54] (p. 498):

$$\frac{dM}{dt}=\left(k_{1}\times \left(1-D\right)+k_{2}\times D\right)\times \left(k_{3}-M\right)-M\times \left(k_{4}\times \left(1-W\right)+k_{5}\times W\right)$$

$$\frac{dD}{dt}=k_{6}\times \left(\frac{M\times \left(1-D\right)}{k_{7}+1-D}-\frac{k_{8}\times D}{k_{9}+D}\right)$$

$$\frac{dW}{dt}=k_{10}\times \left(-\frac{M\times W}{k_{11}+W}+\frac{k_{12}\times \left(1-W\right)}{k_{13}+1-W}\right)$$

In these dimensionless equations the variables *M*, *D*, and *W* represent the concentrations of the three interacting proteins that change over time. Their final values to be calculated by the model are the explanandum, i.e., they must agree with the actual concentrations of the three proteins, the value of which was measured experimentally at the end of a certain real process that took place in the frog egg. The thirteen quantities *k*_1_ to *k*_13_ are the control parameters whose constant value was determined by the modelers and therefore cannot be varied by the calculated system dynamics during the computer simulation. In other words, the self-organization of this small system of three dynamical quantities (variables) requires the determination of thirteen static quantities (control parameters). A few years ago van Hoek suggested a metabolic pathway model for the behavior of the bacterium *Escherichia coli*. Following the same methodology as that of the authors presented above, he used ten coupled differential equations that calculated the values of the same number of variables for the solution of which he used 58 control parameters [48] (pp. 18–20, 45–47). In the last few decades several research groups have carried out computer simulations of whole cells. The model consisting of the three equations introduced above is part of a larger model of the yeast cell cycle that operates with differential equations and was published in the same article [54]. In this model the yeast cell is reduced to 36 state variables, for the computation of which the model makers use 143 control parameters. Thus, on average, for the computation of one variable they use four control parameters. In 2012, a large so-called ‘whole-cell’ model of the cell cycle of a bacterium was published by researchers at Stanford University, in which the behavior of several hundred variables was simulated by specifying 1900 control parameters [45] (p. 389). An essential common feature of all systems biology models known to me that operate with differential equations is that the number of control parameters exceeds the number of variables many times over [46,47,48,49,50,51,52,53,54].

It is well known that in real organisms “a huge number of parameters can significantly modulate a biological process toward very different outcomes in an utterly unpredictable way” [22] (p. 7). This is also reflected in the models of self-organized behavior, in which different sets of control parameters generate different forms of trajectories that, in dependence of the initial conditions, may be directed toward one or more final states or oscillate around a particular area of the state space [55] (p. 52). In systems biological modeling some control parameters represent the degree of activity of specific molecules, while others represent the rate coefficient of reactions in chemical kinetics. Other control parameters represent constraints, such as the boundary conditions of the system—in other words, they represent the matter and energy that the system imports and exports. Control parameters are either constants or entail many constants the value of which cannot be varied by the model’s own dynamics. Control parameters are preset by the model makers and are held constant during a computer simulation. Of course, with each new simulation, the modelers can vary the values of the parameters. In other words, *control parameters are externally fixed factors that cannot be varied by the system’s own dynamics during a computer simulation*. The reason for this is that those quantities canalize the development of the time-dependent variables so that they are logical presuppositions of the system’s possible dynamics. In more philosophical terms, the control parameters are an important part of the explanans. In biological modeling, control parameters are either experimentally derived or estimated or simply taken from the literature [56] (pp. 146–173): “The parameter values (e.g., rate constants in biochemical reactions) can either be determined from biochemical binding experiments (characterizing interactions between individual molecules) or the values of parameters are estimated from time course data. In either case, when it comes to the validation of such models, matching simulations of the model with data, quantitative time series are required” [57] (p. 252). It is a common praxis in systems biology to estimate control parameters “by fitting them to limited experimental data” [43] (p. 986); [58] (p. 60) in order to “[minimize the] distance in data space between the observed data and the model” [59] (p. 3). In other words, in many cases “[p]arameter values […] are estimated by fitting solutions of the equations to a suite of experimental observations […] Common practice among modelers is to ‘twiddle’ the parameters, in order to bring numerical solutions of the equations in reasonable agreement with a set of experimental observations […] If no amount of twiddling can bring the model into agreement with the data, then the modeler must consider changing the governing equations and starts the parameter twiddling process anew” [58] (pp. 48–49). The phenomenon of so-called ‘sloppiness,’ to which much attention has been paid in recent years, however, makes it unnecessary to adapt all parameters to experimental data. In ‘sloppy’ models useful predictions can be made without a precise knowledge of any single parameter [59] (p. 9). Nonetheless, “[t]his does not mean that anything goes; […] educated guesses [must be] made for most parameters, and a remaining handful is fit to the data. […] fitting *m* parameters will be enough to locate the sloppy subspace. […] The information provided by fitting these *m* parameters is often enough to make sharp predictions in similar experimental contexts. Note that this is not true for all possible experiments: in general, the relevant information depends on the questions being asked” [59] (p. 9). This statement makes it clear that even if they deal with ‘sloppy’ models the modelers still have to determine the values of the control parameters in order to minimize the distance between the experimentally observed data and the data calculated by their model.

But the last quotation also leads to a further discussion about the ontological status of both the models and the mathematical mechanisms employed in them. If “the relevant information depends on the questions being asked”, as the authors say, then the researcher’s perspective is crucial for the model and thus also for the parameters it contains. This position is supported by the following statement by two systems biologists: “Mechanistic models, using representations from the theory of dynamical systems, are usually constructed to fit the particular experimental context. Taken together, in the current practice of Systems Biology, the data available largely depend on the technologies and resources available. The choice of the experimental setup, the choice of the model organism, cell line and the choice of technologies used to generate data, usually define a narrow context for which an appropriate modeling approach has to be chosen” [57] (p. 252). This statement is in full agreement with some of the ideas of one of the leading proponents of the New Mechanical Philosophy: Carl F. Craver has supported a subjectivist version of the mechanistic explanation that emphasizes the decisive role of the researchers’ perspectives, i.e., their scientific interests and goals, in determining which parts and activities of a cell or organism are considered constitutive parts of a mechanism that explains a function of interest, and which parts are ignored [60] (pp. 141–143, 155). The constructivity of models, which are based on the interests pursued by certain researchers, is also reflected in the fact that sometimes new, simpler models are derived from original models. Transtrum and his co-authors report on the reduction of an initial model consisting of 29 differential equations and 48 parameters to a model consisting of 6 differential equations and 12 parameters [59] (p. 6). In such procedures, however, “algorithms often evaporate those parameters associated with less prominent features of the data” [59] (p. 6). The constructivity of control parameters, which sometimes results in some of them being eliminated, indicates that the control parameters that will be incorporated into the final model, while empirically adjusted factors often do not correspond to actual facts in real organisms [61] (p. 269).

Both the dependence of models on the subjective perspective of the researchers and the diverse ways in which control parameters are constructed indicate that systems biology modeling has a much higher heuristic than ontological value. In fact, most systems biologists emphasize the heuristic value of their work in developing new biotechnologies and new drugs to treat diseases such as cancer, Parkinson’s, Alzheimer’s, and others. Close to this position is Nicholson’s appeal not to view mechanisms as real things, but as concepts that have only heuristic value for scientific research [62] (pp. 154, 158f., 162). But if that is correct, the question arises, why is there talk of a “systems-ontological approach” in the title of this section? The presence of this expression in the title is justified by the *implicit* and *unconscious* ontologization of systems-theoretical access to organisms by most systems biologists and scientists in general. How else can one interpret the fact that organisms are often treated as if they could be nothing more than dynamic systems of nonlinear molecular interactions? This development has some clearly philosophical overtones, as the biologists who pursue it attribute to the theory of self-organized dynamic systems-ontological and not just heuristic relevance for biology. The most blatant cases of such ontologization, but not the only ones, of which this author is aware, are Lewis Wolpert’s conviction of the computability of the embryogenesis of a multicellular organism and the vision of whole brain emulation pursued by some leading computer scientists [63,64]. Such an ontologization of systems biological mechanisms is not only unjustified, but also highly misleading, especially since in the opinion of this author real organisms obey a completely different logic than our scientific mechanisms, as it will be claimed in Section 3 of this study.

The question of whether systems biology models are assigned a heuristic or ontological value is less important for the present study than the realization that all these models have their most important property in common: They are based on the same implicit assumption about the role of various causal factors in the dynamics of biological systems, as shown below. The term *causal factors* refers to all factors of a model that contribute to the calculation of a system’s dynamics, i.e., variables, control parameters, and equations. In the following, the general term ‘factors’ is used in the sense of ‘causal factors.’ In formal models, which are used in both physics and systems biology, two essentially different types of factors can be distinguished: intrinsic and extrinsic. *Intrinsic factors* of formal models are those factors, which are generated by the system’s dynamics itself. They are the time-dependent values of the variables. *Extrinsic factors* of formal models include all the factors that contribute to the generation of intrinsic factors but are *not* influenced by the system’s dynamics, i.e., the respective state of the system. Control parameters, since they are constants, and initial conditions, which are the values of the variables at the start of a computer simulation, are extrinsic factors—but not the only ones, as we will see shortly. In the above system of equations, the changing values of *M*, *D*, and *W* are the only intrinsic factors and the control parameters k_1_ to k_13_ are the only extrinsic factors. It is obvious that in the present study the terms ‘intrinsic’ and ‘extrinsic’ do not refer to spatial relationships, but rather mean ‘dependent upon dynamics’ and ‘independent of dynamics.’

In the formalisms of systems biology, the most complex factors are the *differential equations* or *the systems of coupled differential equations* themselves (e.g., all three equations of the model described above) that determine the variation of the variables. Those systems of equations are relationships between the less complex factors, i.e., the variables, the control parameters, and the initial conditions. In contemporary formalisms, the systems of equations are not influenced by the changes of the state of the system. They are independent of the dynamics of the system, which clearly qualifies them as extrinsic factors. As relations between simpler factors they can be characterized as *second-order extrinsic factors*. Analogously, variables can be understood as *first-order intrinsic factors* and control parameters as *first-order extrinsic factors*. A system of coupled differential equations such as the one presented above is a single indivisible second-order extrinsic factor.

The essence of the systems-ontological consideration of the nature of the organism can be condensed in two statements that reflect the *logic of contemporary systems* *biological mechanisms*:

(i) In the mathematical mechanisms employed in the models of systems biology, the number of first-order extrinsic factors exceeds several times the number of the first-order intrinsic factors.
(ii) The second-order factor of these mechanisms is always an extrinsic factor.

#### 2.2.3. Process Ontological Approaches

New ontologies, which can be termed ‘process ontologies,’ are currently being introduced into the theory of the organism and the philosophical consideration of biological questions in general. They are based on the hypothesis that the ultimate elements of physical reality are processes, while at the same time introducing a philosophically elaborated definition of the term ‘process’ that differs significantly from the meaning, which is ascribed to this word in both its current scientific and everyday language usage. The well-known version of process ontology originated in the writings of Alfred North Whitehead (1861–1947) in the early 20th century, but was clearly anticipated in the works of William James, Henri Bergson, and Charles Sanders Peirce. At present, however, another form of process ontology is finding its way into biology: In their programmatic essay “A Manifesto for a Processual Philosophy of Biology”, John Dupré and Daniel J. Nicholson deny Whitehead’s ontology, as presented in his magnum opus *Process and Reality* and in his later works, almost any biological relevance because, as they claim, his thought would be based on panpsychistic and theological arguments and besides his language would be opaque [65] (pp. 7–13). Instead they propose an essentially different process ontology, which they consider compatible with the worldview of contemporary biosciences. However, although they refuse the occupation of biological thought by the concept of mechanism [62], which is so influential in systems-theoretical biology, in the opinion of this author they actually created another variant of the systems ontology that is expressed through process vocabulary. For this reason, and because, as it will be claimed in Section 3 of this study, organisms obey an essentially different logic than that of systems ontology, the focus in what follows is on Whiteheadian process ontology.

Whitehead is often regarded as one the most original innovators of the 20th century philosophy of nature and metaphysics. One basic premise of Whitehead’s metaphysics is that all elementary actual beings—of which the enduring micro-, meso-, and macroscopic entities of the physical universe consist—are indivisible processes, which he calls ‘actual occasions.’ They are conceived as short-lived, indivisible (atomic) processes whose spatiotemporal expansion covers a broad spectrum, from quantum events to macroscopic processes that take place in the brain and are equipped with consciousness. Hence, the most basic real beings that make up the physical world are not considered substances, neither in the sense of Aristotle nor in the sense of Descartes, but indivisible processes. Whitehead introduced his understanding of ‘process’ against the background of all versions of mechanistic thought that were influential in the first half of the 20th century. The conception of the ‘actual occasion’ is based on three main pillars: First, any ‘actual occasion’ is to a certain degree a creative being, since it “functions in respect to its own determination” [66] (p. 25). As being directed toward their own self-determination, ‘actual occasions’ exhibit final-state-directedness. Thus, they may be considered teleologically self-building beings whose final constitution is not preformed in them but emerges gradually as an actualization of intrinsic tendencies. Second, ‘actual occasions’ are indissolubly related to a specific environment [66] (pp. 73, 77, 80). This idea corresponds exactly to what in metaphysics is called ‘internal relations’—meaning relations, without which neither of the related beings could physically exist (nor could their essential features be understood). Third, ‘actual occasions’ are conceived of as indissolubly mental-physical beings: “Each actuality is essentially bipolar, physical and mental, and the physical inheritance is essentially accompanied by a conceptual reaction [...] always introducing emphasis, valuation, and purpose” [66] (p. 108). Due to their mental pole ‘actual occasions’ possess an elementary subjectivity.

According to this ontology, everything that lasts in time consists of ‘actual occasions,’ consequently living beings are understood as coherent groups of internally related mental-physical processes that are teleologically coordinated elementary subjects [66] (p. 103).

Whiteheadian ontology has so far had a limited impact on orthodox biological thinking. Nevertheless, in the early 20th century he had a strong influence on some well-known biologists. His influence on biological thinking has increased again in the last few decades. The eminent British theoretical biologist and philosopher of biology Joseph Henry Woodger (1894–1981) was heavily influenced by Whitehead when he published his particularly influential work *Biological Principles* (1929). Inspired by Whitehead’s space-time theory, he regards an organism as a four-dimensional event, the temporal dimension of which contains its spatial parts [67] (p. 301f.). Woodger applies various ideas from Whitehead to biological questions, such as the idea of the internal relationality of spatiotemporal relationships on embryogenesis and evolution [67] (pp. 354, 421). The Anglo-Australian zoologist Wilfred Eade Agar (1882–1951) related central ideas of the mature process ontological phase of Whitehead to fundamental biological questions. Following Whitehead, he conceives of organisms as subjects or nexūs of subjects [68] (pp. 7, 14f., 57). He also considers cell organelles, cells, tissues, and, in animals, the ‘central agent,’ which is correlated with the central nervous system, as subjects and active agents [68] (p. 12). In his general theory of biology, the Canadian-American biologist Ralph Stayner Lillie (1875–1952) shares with Whitehead the conviction that an agent operates in every living being, which counteracts the growth of entropy and has both physical and mental aspects [69] (pp. 176, 191f.). The psychophysical nature of all organisms, which cannot be reduced to physical mechanisms [69] (p. 178f.), ensures their physical stability [69] (p. 140). British embryologist and geneticist Conrad Hall Waddington (1905–1975) is often *mistakenly* considered to be the biologist most influenced by Whitehead. One of his most important achievements is the systems-theoretical description of the activity of genes in embryonic development [70] (pp. 47, 51–56). His description of embryogenesis is entirely compatible with the systems-theoretical materialism of modern systems biology, of which he is a co-founder, and shows—in sharp contrast to the works of Agar and Lillie, who, unlike Waddington, emphasize the subjectivity of organisms and cells—no reference to Whitehead’s mature process ontology [70] (pp. 40–56). Rather, he admits a clear distance to Whitehead [71] (p. 144). Waddington is clearly a pioneer of the cybernetic/systems-theoretical realignment of biological thinking that dominates modern theoretical biology and systems biology [71] (p. 143f.). The criticism of these disciplines from a Whiteheadian perspective [2] (pp. 122–334) [3,23] affects his work equally. Some of Whitehead’s insights also flow into the biologically relevant ‘systems philosophy’ of the Hungarian systems-theorist Ervin Laszlo, in which he merges ideas from von Bertalanffy’s general systems-theory with basic cybernetic ideas [72]. The Australian geneticist Charles Louis Birch (1918–2009) made a special contribution to the dissemination of Whitehead’s ideas in biology. Following Whitehead, he considers orthodox biology to be mechanistic thinking dominated by physics, the limitations of which (the mechanistic thinking) are revealed in particular by the emergence of life and consciousness [73]. He shows that process-philosophical ideas—e.g., the idea that electrons, atoms, and biological cells are subjects correlated with each other through internal relations—can help overcome mechanistic thinking in physiology, behavioral science, sociobiology, genetics, developmental biology, and evolutionary theory [74]. He sees no reason to reduce subjectivity to the function of nervous systems [73] (p. 15). Birch sees the essence of life in the teleological introduction of novelty and in overcoming the forms of causality studied by physics [75] (p. 109). The British mathematician and physicist Roger Penrose and the American physician Stuart Hameroff introduced a quantum theory of consciousness, inspired by Whitehead’s process ontology. They think that coherent quantum oscillations arise in an area of the brain and spontaneously collapse. Hameroff interprets the process of the collapse of quantum coherence in terms of a Whiteheadian ‘actual occasion’ of macroscopic size, which can extend over tens of thousands of neurons [76] (p. 11), [77] (p. 49 f.). The subjective side of such processes constitutes the interiority of our acts of consciousness. The American physicist Henry Stapp also deals with the problem of the internally caused collapse of a quantum entity to a space-time datum. He attributes a decisive role to human thoughts in reducing the quantum coherence of the brain [78] (p. 199). Stapp considers the material manifestation of a macroscopic quantum event in the brain to be the physical side of a Whiteheadian ‘actual occasion,’ the mental side of which is a thought [78] (pp. 204–211). Based on Whitehead’s process ontology, the Austrian biologists Gernot and Renate Falkner proposed a processual biology. They think that living beings recreate their structure every time they experience an environmental change in order to adapt to their new environment. This creates new structural elements that are coordinated with one another in functional harmony [79]. Finally, this author proposed a new process ontological biophilosophy, the main philosophical pillars of which are the ontologies of Whitehead and Bergson [2]. One of its goals is to overcome the systems-theoretical reduction of organisms [2,3]. By synthesizing the theory of the self-organization of dynamic nonlinear systems and quantum biology on the one hand with Whitehead’s process ontology on the other hand, an extension of the basic ideas of Whitehead’s biophilosophy is proposed. According to the new approach, both organismic self-preservation and embryogenesis are guided by a particularly complex form of ‘actual occasions’ [2] (pp. 575–630), [3]. Finally, it should be mentioned that important process theologians have published insightful articles on the biological relevance of Whitehead, of which only a few can be mentioned here [80,81,82,83].

## 3. The Logic of the Organism

We can also apply to the causal factors that determine the dynamics of organisms the two distinctions between intrinsic and extrinsic factors and the first and second order factors proposed in the presentation of the logic of systems biological formalisms. This requires that the terms ‘intrinsic’ and ‘extrinsic’ are not interpreted as ‘internal’ and ‘external’ respectively in the spatial sense of both words but as ‘dependent upon dynamics’ and ‘independent of dynamics’ respectively, as suggested above.

The logic of the organism as introduced here has two main dimensions. The first one is based on the concept of first-order factors. The term ‘first-order intrinsic factors’ refers to all material and energetic quantities generated by an organism that have a causal effect on its dynamics. This category includes quantities within the living body, such as the concentration of regulatory proteins, scleroproteins, hormones, ATP molecules, etc., as well as particular environmental factors such as regulated humidity and room temperature, which individual organisms or groups of organisms control through a corresponding niche construction in order to improve their living conditions. The term ‘first-order extrinsic factors’ refers to all factors that exert a causal influence on the dynamics of an organism, but are not influenced by the latter. This category includes fundamental laws of nature that determine physicochemical processes, initial conditions, such as the parental genetic constitution, the physicochemical environment of a zygote at the time of its fertilization, and environmental conditions that cannot be changed by organismic activity, such as gravitation, radioactivity, geological processes, solar activity, and the forms and quantities of available energy and matter. However, one of the most essential characteristics of biological evolution is that the borderline between first-order intrinsic and extrinsic factors is fluent. Especially during the evolution of human intelligence, some of the extrinsic environmental factors just mentioned have been transformed into intrinsic ones. Good examples of such transformation are agriculture and livestock farming, through which human societies determine the type, quantity, and quality of the food resources available in their environment, as well as nuclear technology, which has massively changed the level of radioactivity in some human environments, with devastating consequences. And if we believe some science fiction authors, we can even speculate about civilizations that control the gravity of their planets, the radiation of their suns, and even manipulate laws of physics. The other essential dimension of the logic of the organism is based on the idea of the second order factors, which also applies to organisms, as we shall see in a moment. Nonetheless, although the distinction between first-order and second-order factors on the one hand and between intrinsic and extrinsic factors on the other hand applies to organisms, *real organisms do not obey the logic of systems biological mechanisms*. This essential difference, which is of the utmost importance for the logic of the organismic mode of being, has two reasons.

First, in sharp contrast to systems biological formalisms, organisms are able to change the value of most quantities that in systems biological models are represented by parameters, i.e., fixed quantities that represent, for example, rate coefficients or boundary conditions. As already mentioned, current research shows that both unicellular organisms and cells of multicellular organisms are able to specifically control the course of their intracellular molecular reactions through various modifications of their own material structure: The binding affinities between single biomolecules (e.g., protein or DNA) to their ligands (e.g., drug or inhibitor) can be altered by the surrounding complexes of proteins, lipids, and cellular substructures, thus allowing the cells to regulate the kinetic rates of particular intracellular biochemical reactions. The intensity of the correlation between elements of biological networks, such as genetic and metabolic networks, is quantified by “kinetic-like weights […] but it is well known that in physiological settings these weights can vary of orders of magnitude” [20] (p. 2). As a result, the kinetic control parameters of biological networks are modifiable: “[K]inetic parameters in biological networks are not hard-wired in the network architecture and can be relatively easily circumvented. There are in fact some experimental data demonstrating the possibility of many orders of magnitudes variations of kinetic parameters of biochemical reactions” [20] (pp. 3–4). Of course, those massive variations do not occur coincidentally but are controlled by the cells themselves. It is also a fact that a cell “can greatly enhance the targeting of a molecule through modification of its [the cell’s (S.K.)] dimensionality” [21] (p. 35). The regulation of the architecture of its own cytoskeleton enables the cell to influence intracellular enzymatic reactions [21] (p. 35). These and other contemporary findings support this author’s assumption that real organisms are capable of varying the values of most of the quantities that in systems biological mechanisms are represented by fixed parameters. This means, however, that, in contrast to contemporary biological formalisms, in real organisms the number of extrinsic factors of the first order is only a tiny fraction of the number of all dynamic quantities that mutually determine their development. Hence, the first rule of the logic of the organism is as follows: (i) *In real organisms the number of first-order intrinsic factors always exceeds by many times the number of the first-order extrinsic ones*.

Second, during the growth and regeneration of unicellular and multicellular organisms and in the embryogenesis of the latter, a variety of new types of proteins are synthesized and major morphological and physiological changes take place. Radical targeted configurations of the morphology of living beings also take place at the cellular level. As already mentioned, there are good reasons to assume that cells govern their metabolic and functional processes through the modification of their own shape: “[B]iological functions are shaped by the three-dimensional context in which they occur. In fact, an impressive body of evidence demonstrated that by physically modifying the shape of a cell, we can trigger a bewildering number of different outputs: from differentiation to apoptosis, including even reversion of a cancerous phenotype” [22] (p. 5). This is due to the fact that the spatial structure of the three-dimensional context, within which chemical processes and biological functions occur, “literally bends the interacting components and their dynamics, leading to dramatic increases in the efficiency of signal transfer as well as to enhanced specificity of signal flow” [22] (p. 5). Therefore, any “modification of the system’s architecture can canalize the biological processes in a very different way”, which actually means that “an unexpected level of causality emerges uniquely from the specific configuration adopted by the system” [22] (p. 5). It is also well known that in order to adapt to external forces, such as shear and tensile stress; compressive forces; and hydrostatic pressure, cells modify their behavior and remodel their shape “through actomyosin- and cytoskeletal-dependent modifications”, which in turn allows them to “exert a reciprocal influence on their microenvironment (mechano-reciprocity), as well as on gene expression” [21] (p. 36). Living cells generate active tension in their cytoskeleton, so that, by “altering the balance of forces transmitted across the adhesion site, the signaling machinery can be altered, thereby producing different functional outputs” [21] (p. 36). Embryonic processes display an even more radical intrinsicness of the modification of an organism’s morphological, physiological, and biochemical structure. Those examples mean that the network of causal relationships within a real organism is subject to change. As a result, the material structure of an organism is a sequence of permanently generated new relationships between its own first-order intrinsic and first-order extrinsic factors. As mentioned above, in contemporary formalisms, those relationships are represented by fixed systems of invariable differential equations. Since these systems of equations are static, they are second-order extrinsic factors. In contrast, in a primitive unicellular or mature multicellular organism, even if the latter does not undergo a process of radical regeneration or readaptation, the relationship between both kinds of first-order factors is itself an intrinsic factor. This is the case, since, on the one hand, that relationship is permanently determined by the organism’s dynamics and, on the other hand, it canalizes these dynamics. The relationship between a living being’s first-order intrinsic and extrinsic factors constitutes its second-order factor. Thus, if that relationship is an intrinsic one so is the organism’s second order factor. Consequently, a system of differential equations could only then represent the dynamics of an embryo or the cell cycle with a high accuracy if it were subjected to a continuous radical transformation. This is particularly necessary because both during embryogenesis and during the cell cycle, new types of molecules are synthesized which have to be represented by new variables. This would require the creation of new equations and the variation of the old ones. The permanent transformation of the formal system of equations would continue until one of the forms it takes on calculates the final state of the respective process. These facts about the radical morphological and other restructuring of individual cells and multicellular organisms are summarized by the second rule of the logic of the organism: (ii) *In a real organism, the second-order factor is necessarily an intrinsic factors* or, in other words, *there is no second-order extrinsic factor in a real organism*.

Both rules of the logic of the organism are essentially connected with one another. Since all first order factors of an organism constitute a single causal network, the organism would not be able to harmonize the various radical changes of some of these factors with one another if its structure were not a second-order intrinsic factor and were thus able to reorganize itself.

The logic of the organism actually constitutes an even more abstract level of analysis of the organismic phenomenality than the three levels exemplified in Section 2.1. Accordingly, it does not explain the organismic phenomenon but only describes it. Therefore, as said above, it occupies a position between the phenomenal and the causal-ontological analysis.

### 3.1. The Second-Order Intrinsic Factor Is a Self

To assert the intrinsicness of an organism’s second-order factor means that the organism is able to autonomously regulate the relationship between its intrinsic and extrinsic first-order factors, that is, to vary that relationship teleologically or to stabilize it against external perturbations. Since both types of first-order factors involve some environmental conditions, the intrinsicness of the second-order factor enables the organism to autonomously interact with its immediate environment and to determine its own boundary conditions. A breathing and self-nourishing organism is a being that controls essential boundary conditions autonomously.

In addition, second-order intrinsicness gives many organisms a remarkable flexibility, which enables them not only to teleologically interact with their environment in a suitable manner and successfully master environmental challenges through physiological or even morphological transformations, but also to subject their immediate environment to massive changes. This is in a sharp contrast to systems studied by physics, including self-organized systems, also called ‘dissipative structures’, the spatial borders of which and their border conditions are determined either by a human experimenter or by an external physical factor, including chance. The term ‘second-order intrinsic factor’ designates the most fundamental organizing principle of any living being: Most aspects of the material and energetic constitution of an organism serve the autonomous maintenance and perpetuation of its self-organized structure. In a previous co-authored article, this author suggests that “a dynamical process organized in such a way that it minimizes the probability that its organization will be lost” may be labeled a *self* [84] (p. 417). A self is a process that reinforces the synergistic relationship between its elements. The organismic type of selfhood is characterized by the fact that the material structure not only builds itself up—inorganic self-organized systems do this too—, but above all autonomously generates boundary conditions and internal conditions (both are first-order intrinsic factors) that are necessary for the further maintenance of the organism. Only on the basis of this specifically biological understanding of selfhood can a second-order intrinsic factor be referred to as a ‘self’.

The organismic self is “a form of individuality” [85] (p. 309) because in any second-order intrinsic factor the related first-order intrinsic and first-order extrinsic factors become inextricably causally and functionally interweaved so that the whole self-determining process may not be physically divided into more elementary processes [85] (p. 469).

At this point the objection could be raised that the indissoluble physiological-functional integration is a necessary but not a sufficient condition for biological individuation. Cells, organisms, and colonies are physiologically-functionally integrated, so one may wonder, which of them is the true biological individual? As Pradeu says, “[e]ach zooid [of the colonial organism *Botrylus schlosseri* (S.K.)] has a membrane and is, at least to some extent, an integrated whole, but one could say that the true functional integration happens at the level of the colony, which has a common vascular network” [10] (p. 252). Pradeu claims that the sufficient criterion for biological individuality must be sought in the field of immunology, since the immune system determines what belongs to an organism and what does not. In other words, “Immunity offers a criterion […] for what makes the organism a *unit* constituted of different entities through time” [10] (p. 253f.). Since all multicellular and unicellular organisms have immunity, immunity is also ubiquitous and can therefore be the basis for a theory of organismic individuation [10] (p. 255). An immunological definition of the organism can be formulated from this argument: “An organism is a functionally integrated whole, made up of heterogeneous constituents that are locally interconnected by strong biochemical interactions and controlled by systemic immune interactions that repeat constantly at the same medium intensity” [10] (p. 258). This immunological approach to the concept of the organism is undoubtedly justified. Against the background of the logic of the organism, however, it must be assumed that the immune system can only function properly because both the immune cells and the entire organism obey the two rules of said logic. Above all, this means that the host organism and each of its immune cells, as well as each of its other cells, are controlled by their own second-order intrinsic factors. Consequently, their organization goes beyond the logic of systems biological mechanisms.

The question of whether the most fundamental biological individuals or selves are cells, organisms, or colonies is definitely a core question in biology. But this article does not address this issue. Instead, it provides arguments that every biological self, regardless of the category to which it belongs, has the organization of a second-order intrinsic factor. In this essay the attempt is made to introduce abstract rules of a logic that captures the radical autonomy of the organismic mode of being. However, this logic can also apply to biological entities that are not organisms. It can apply, for example, to particular colonies, such as *Botrylus schlosseri,* or to symbiotic wholes that can also manifest biological selfhood. This does not limit its validity for the organism, because it is a necessary but not a sufficient condition for something to be an organism.

### 3.2. The Second-Order Intrinsic Factor Acts in an Umwelt

The identification of biological selves is also complicated by the fact that there is a “critical but contingent relationship between selves and physical boundaries” [85] (p. 471). Since any living being maintains itself through a selective exchange with its environment, any adequate theory of organismic selfhood and individuality must necessarily be an organism-environment theory. This statement, self-evident at first glance, conceals a serious problem, namely the question of the relationship between the individuality of an entity and its embedding in the environment. One of the problems this question raises is the nature of the processes by which the entity delimits itself from the surrounding spatial continuum when the former exchanges energy and matter with the latter. Organismic selves or second-order intrinsic factors establish their relationships with their environment by autonomously selecting certain environmental facts relevant to their biological needs and interacting with them. As will be shown below, this statement, which is trivial for all non-Darwinian biologists, puzzles those biologists and philosophers of biology who believe that between the causal orders that prevail in organisms and in inorganic self-organized systems there is only a difference in degree and not in kind. Nicholson’s non-Whiteheadian process ontological understanding of organism supports this erroneous assumption [86] (pp. 144f., 151f.).

In 1909 Jakob von Uexküll introduced the term ‘Umwelt’ referring to those features of a living being’s environment that are meaningful to it [87] (pp. 118, 249, 252f.). Therefore ‘Umwelt’ may be translated as ‘meaningful environment’ [88] (p. 268).

In contrast to organisms, entities of inorganic nature do not have an ‘Umwelt’ but just an ‘Umgebung,’ to use another term from von Uexküll, with which he referred to the mere physical surroundings of an entity. This applies also to nonlinear dynamic systems studied in theory of self-organization, such as flames, whirlpools, Bénard convection cells, the Great Red Spot of Jupiter, granules on the sun’s surface, earth mantle convection, reaction-diffusion systems (e.g., Belousov-Zhabotinsky and similar reactions), soliton waves, and complex atmospheric phenomena. The self-organization of such systems constitutes only a reaction to energetic and/or material gradients that existed in the spatial surroundings of the systems before the latter emerged. The systems are directed toward the increase of the production of entropy (but not its maximization, as it is often wrongly claimed) and thus more efficiently deplete the gradients, which are the condition of their existence. This means, however, that inorganic self-organized systems of today’s physics are *self-undermining* processes. In contrast, organisms organize themselves toward the temporal extension of their existence and, as biologists Gernot and Renate Falkner have shown, although organisms operate far from the thermodynamic equilibrium, their processes of physiological adaptation succeed in *reducing* their entropy production [89] (pp. 81, 89, 93).

These essential differences between organisms and inorganic self-organized systems have serious consequences for the better understanding of the organismic mode of being. Systems studied in physics of self-organization are patterns, the spatial boundaries of which emerge within a physical continuum as a subsequent reaction to the inhomogeneity of this continuum. The first condition of inorganic self-organization is the inhomogeneity of a continuum of matter and energy, i.e., the existence of material-energetic gradients. As a reaction to this initial inhomogeneity, several spatiotemporal patterns of activity subsequently emerge within the continuum, which, in the words of von Uexküll, is their ‘Umgebung.’ These patterns have visible spatial boundaries that give the impression that they are self-sustaining individuals. In reality, they are just internal restructurings of the continuum that surrounds them. In contrast, organisms exhibit another relation between continuity and individuality: They do not simply react to gradients in their surroundings but interact with their Umwelt in ways that support their self-perpetuation. As a result, the spatial limitation of organisms is generated by a process of self-limitation that cannot be captured by contemporary physics of self-organization. The spatial borders of inorganic self-organized systems, such as snowflakes, whirlpools, twisters, and the Great Red Spot of Jupiter, constitute an internal reorganization of an energetic-material continuum, which is an internal differentiation of that continuum. However, since this reorganization increases the exhaustion of the energetic-material gradients of the continuum, it ultimately *increases the speed of the destruction* of the continuum’s own work potential. In other words, inorganic self-organized systems are non-autonomous internal parts of the overall restructuring of the continuum. They are *forced* by their dominant surrounding (‘Umgebung’) to emerge and persist until the gradients within their surrounding continuum are exhausted. Metaphorically speaking, inorganic self-organized systems are internal affairs of the material-energetic continuum.

In sharp contrast to systems studied by physics of self-organization, organisms are processes whose self-limitation reveals their autonomous interaction with the physical continuum that surrounds them. They do not simply react to externally and extrinsically imposed border conditions that remove them from thermodynamic equilibrium. Rather they actively remove themselves from this equilibrium by autonomously determining essential boundary conditions and, if necessary, other first order intrinsic factors as well. Their autonomy can be illuminated by the two main rules of the logic of the organism, a logic of a type of processuality unknown to contemporary physics of self-organization. There are good reasons to assume that organismic self-organization is a phenomenon that, for fundamental reasons, eludes the methodology of the entire body of physics. This topic is outside the focus of this article. The following note will suffice here: In a recent article Montévil shows that the relationship between equations, variables, border and initial conditions, and control parameters in all areas of physics obeys a logic that cannot be followed by sciences, the objects of which have an irreducible historicity. This clearly applies to biology, since organisms have evolved over billions of years [90].

The position exposed here is ideally summarized by George Canguilhem, who bases his own considerations on the completely different role of the milieu in physics and biology on von Uexküll’s theory [19] (pp. 110–120): “A center does not resolve into its environment. A living being is not reducible to a crossroads of influences. From this stems the insufficiency of any biology that, in complete submission to the spirit of the physico-chemical sciences, would seek to eliminate all consideration of sense from its domain. From the biological and psychological point of view, a sense is an appreciation of values in relation to a need. And for the one who experiences and lives it, a need is an irreducible, and thereby absolute, system of reference” [19] (p. 120).

Since the organism’s creation of a self-other boundary involves a representation of its Umwelt in relation to its needs, Umwelt and self are two sides of the same coin. In order to satisfy its needs, the autonomous self is forced to interact with various types of entities within its Umwelt that are in a certain sense external but not extrinsic to it.

At this point the question arises as to whether the word ‘external’ is to be understood spatially or functionally. The first understanding of ‘external’ corresponds to the morphological access to the organism and the second to the physiological-functional (see Section 2.1.2). In the first case, the boundary between the organism and its Umwelt coincides with the morphological boundaries of the organism, so that the Umwelt begins on the outside of the organism’s visible surface (skin, membrane etc.). In the second case, the said boundary is determined by the physiology of the organism and is therefore invisible to the layman. The physiological-functional perspective can be decisively supported by Pradeu’s immunological definition of the organism, which was introduced above. He rejects the position “that the organism is that which is behind the skin (or any membrane)”, so that “intestinal bacteria, bacteria on the skin, long-tolerated parasites, etc.” are to be regarded as part of the organism and not as part of its Umwelt [10] (p. 261). Conversely, from an immunological-physiological point of view, biological entities in the organism’s blood, intestine, lungs, etc., which are fought by its immune system, are to be regarded as parts of the organism’s Umwelt.

Obviously, the underlying understanding of the Umwelt depends on the theoretical approach to the concepts of organism and self. From the perspective of the logic of the organism proposed here, however, both possible approaches, the morphological and the physiological-functional, must coincide in one absolutely essential point: The organismic self must in any case be conceived as a second-order intrinsic factor. This underlines the fundamental importance of the latter factor for the concept of the Umwelt, since the organism itself determines which elements of its Umgebung constitute its Umwelt.

### 3.3. The Second-Order Intrinsic Factor Varies Its State Space

The second-order intrinsic factor enables the organism to carry out radical qualitative and quantitative transformations of its material structure. During the cell cycle and the regeneration of unicellular organisms as well as the regeneration, growth, and embryogenesis of multicellular organisms, new sorts of molecules are synthesized. These are new types of first-order intrinsic factors that are to be represented by new variables in the corresponding formalisms. However, this has consequences for the state space, which can accommodate the trajectories that represent the development of the organism: The appearance of new variables requires that the number of dimensions of the corresponding state space be increased. In other words, only continually growing state spaces would be able to represent basic biological processes of unicellular and multicellular organisms. But, as Stewart Kauffman states, we do not have a mathematical framework that can describe a process that autonomously enlarges its own state space, and he concludes that “the way Newton, Einstein, Bohr, and Boltzmann taught us to do science is limited” [91] (p. 136). The correctness of this diagnosis is particularly underlined by the fact that neither the physics nor the mathematics of self-organized dynamic systems deal with processes that change their own state spaces. Thus, another consequence of the intrinsicness of the second-order factor is that the logic of the organism transcends the logic of physics and mathematics of dynamic systems. A satisfactory understanding of the organismic mode of being can only be achieved through a future biology that will be erected around the idea of higher order selfhood—an idea that clearly transcends the current understanding of self within the paradigm of self-organization.

### 3.4. The Logic of the Organism Harmonizes Well with Process Ontology

Whitehead’s concept of ‘actual occasions’ as a description of the basic processes of reality was not explicitly developed for biology. Nevertheless, essential ideas of his process ontology show substantial similarities with the two rules of the logic of the organism proposed above. This may be due in part to the anti-mechanistic stance that the logic of the organism shares with Whitehead’s ontology, which was introduced against the backdrop of all the influential versions of mechanistic thought in the first half of the 20th century.

The essence of the idea of second-order intrinsicness is anticipated in Whitehead’s ontology through the conception of the ‘actual occasion’ as a creative being that “functions in respect to its own determination” [66] (p. 25). Since they are directed toward their own self-determination, ‘actual occasions’ exhibit a special form of final-state-directedness or teleology, which enables them to gradually create the blueprint of their own form in the process of their self-formation. Whitehead’s description of the emergence of an ‘actual occasion’ is comparable to the progressive emergence of intrinsic causal factors in embryogenesis [66] (pp. 240–261). Thus, ‘actual occasions’ can be viewed as teleological selves, the final form of which does not exist in them as a preformed program at the beginning of their self-formation, but rather develops gradually as an actualization of intrinsic tendencies. Teleological self-formation is an essential characteristic of all entities that are ruled by a second-order intrinsic factor. This is particularly demonstrated by the organismic mode of being: As said above, the main common feature of all organisms is that although they change their matter and energy permanently, they retain their form. Or, if they grow or regenerate themselves, change it in a very specific way not reducible to the nature of their material and energetic elements. Organisms are self-stabilizing processes that are not determined by any particular entity or by a particular type of entity, such as genes. Organisms manage to create and maintain their form autonomously because the second-order intrinsicness of their material structure enables them to stabilize themselves by fine-tuning the interactions between their material components. Besides teleological self-formation, ‘actual occasions’ also have another characteristic of biological organisms, which is essentially related to the second-order intrinsicness of the latter: ‘Actual occasions’ require an adequate environment to emerge. During their self-formation, they are closely connected to their environment, which consists of other, already completed ‘actual occasions.’ However, they are not just reactions to extrinsic conditions. In other words, ‘actual occasions’ are not determined by facts, which, according to the above introduced terminology, could be described as ‘extrinsic factors,’ although they also depend on such facts. As they emerge, they add particular relevance to certain features of their environment while making others irrelevant. This means that ‘actual occasions’ are inextricably linked with certain characteristics of their environment, which they select and appropriately include in the process of their self-formation [66] (pp. 73, 77, 80). In this sense, an ‘actual occasion’ can be said to have an Umwelt and not just physical surroundings (‘Umgebung’). This corresponds to Whitehead’s emphasis on the ontological priority of the basic atomic processes of reality over the spatiotemporal continuum: ‘Actual occasions’ are not just individual (atomic) acts that autonomously determine their spatiotemporal boundaries autonomously, but, beyond that, the spatiotemporal continuum surrounding them is generated through their coordinated activities [66] (p. 35).

In addition to the second rule of the logic of the organism, Whitehead’s process ontology also anticipates the first rule in a certain sense. An ‘actual occasion’ begins its process of self-formation with the integration of facts that arose before it appeared, but gradually transforms these initially extrinsic factors into its own intrinsic ones. As the process unfolds and gradually creates the blueprint of its final form, the generated intrinsic factors are repeatedly transformed, integrated and reintegrated into a new synthesis. This corresponds to the first rule of the logic of the organism, which actually states that an organism consists almost exclusively of first-order intrinsic factors, i.e., of entities whose constitution is determined by this organism.

These explanations demonstrate the possibility of abstracting a general process logic from Whitehead’s concept of the ‘actual occasion’ that can serve as a logical basis for a future theory of the organism. Even if one rejects Whitehead’s process ontology (e.g., because of its alleged panpsychism), there is no reason to reject the biological relevance of a process logic that can be extracted from it. The present article is only the beginning of such an intellectual adventure.

## 4. On the New Project of *Biological Statistical Mechanics*: Heuristic Value Meets Ontological Openness

Given the anti-mechanistic spirit of the logic of the organism in this essay, one may wonder why the latter is included in an issue on the new project of Biological Statistical Mechanics. This question would be justified if the word ‘Mechanics’ in the name of the project in question implied an *ontological* meaning, similar to that, which the New Mechanical Philosophy or New Mechanism ascribes to the term ‘mechanism.’ This is, however, not the case. To see the essential differences between Biological Statistical Mechanics and neo-mechanistic approaches in contemporary philosophy of biology, we need to look at the main ideas underlying the new project.

First, Biological Statistical Mechanics aims to study the dynamics of life on all scales of definition, from cell biology to ecology and epidemiology, while also taking into account that the interaction of a multiplicity of scales in both time and space is a fundamental feature of biological entities of all kinds and orders of magnitude. Biological Statistical Mechanics does not essentially differ from statistical mechanics, which investigate how the emergent properties of macroscopic systems (such as temperature and pressure) are related to microscopic state fluctuations. However, instead of being based on order-independent statistics (standard deviation, probability distributions) it is based on order-dependent statistics that quantify the correlation structure of certain quantities. This shift is due to the peculiar nature of biological systems that are characterized by meaningful spatial and temporal correlations among their parts. Due to this peculiarity of biological units, the new approach, in contrast to classical thermodynamics, cannot rely on the reduction of the respective system to macro-parameters such as volume, pressure or energy. Instead, the proponents of the new project must seriously consider the particular correlation structure of the system at hand (e.g., correlation structure of the data set by Principal Component Analysis). This structure is subject to abrupt changes that take place during transitions such as differentiation, morphogenetic development, disease outbreaks, and the destabilization of ecosystems. Since these changes make the correlation structure more visible, the focus on state transitions is considered the most fruitful direction for establishing a Biological Statistical Mechanics. Second, the new approach mediates between the macroscopic examination of an entire organism and the microscopic view of molecular biology. It introduces a new urgently needed *mesoscopic* level of analysis that is able to connect the different levels of investigation. It lies ‘in the middle’ between pure ‘bottom-up’ approaches, according to which the causally relevant layer is the microscopic one, and ‘top-down’ approaches, which attribute causal relevance to the layer, in which general laws are defined. Biological Statistical Mechanics is strongly linked to the generalization of statistical approaches for mesoscopic nonlinear processes that occur far from thermodynamic equilibrium. Third, mesoscopic processes are often related to symmetry changes responsible for critical dynamics of biological transformation. In the statistics and thermodynamics of biological entities, there is a strong relation between symmetry changes, which cause critical transitions during biological transformation, and internal variables [92] (pp. 145–169). Accordingly, the study of symmetry changes could help define meaningful internal variables, which, from the point of view of the logic of the organism, constitute the majority of the first-order intrinsic factors.

Systems of coupled differential equations are widely used in biological modeling. As shown in Section 2.2.2, these systems are characterized by a strict separation between variables and parameters. However, the proponents of Biological Statistical Mechanics are well aware of the fact that the mathematical approach “is severely hampered, in the case of biological systems, by a lot of problems” [20] (p. 2). First of all, “the practical impossibility to attach to the whole set of edges [of a graph represented by a system of equations (S.K.)] reliable kinetic-like weights for quantifying the entity of the between elements correlation” should be mentioned [20] (p. 2). This can be done “[o]nly in the case of very small networks”, namely “by means of the statistical estimation of the parameters from experimental data”, but, as mentioned above, “it is well known that in physiological settings these weights can vary of orders of magnitude” [20] (p. 2). In addition, since many of the biochemical substances and the relationships between them are unknown, in many cases it is not possible to rely on the complete knowledge of the wiring diagram of the network [20] (p. 2). Giuliani recognizes very clearly that the ability of biological entities to vary the value of their kinetic parameters depending on their (the entities) state has enormous consequences for biological thinking: “The plasticity and state dependence of the kinetic parameters of the same network adds *indeterminacy* to the picture and *asks for a statistical perspective*” [20] (p. 11) (italics added). This quote is illuminating for two reasons: First, Giuliani gets to the heart of the problem of the biological sciences: *the irreducible indeterminacy of the living*. Second, he connects the conception of Biological Statistical Mechanics with the idea of indeterminacy, which is an essential characteristic of all types of biological entities. Both insights underline the necessity to proceed carefully when transferring established physical concepts into the biological realm and to avoid a rigorous mathematical description in terms of differential equations.

It is obvious that the proponents of the new project pay close attention to the peculiar nature of biological entities, which significantly differs from the nature of inorganic systems that have been investigated by physics. They are well aware of the failure of strictly deterministic molecular biological approaches to predict the properties of biological entities at the system level. They deal with biological problems without considering them only as an occasion for interesting applications of physical and mathematical concepts. In sharp contrast to many systems biologists, the proponents of Biological Statistical Mechanics do *not* ontologize their mathematical models. Of course, today’s biological modeling can neither do without the strict separation of variables and parameters, nor work with systems of equations that can be varied by their own calculations. These limitations are dictated by the nature of contemporary mathematics and therefore cannot be overcome in the near future. Nevertheless, modeling cannot be dispensed with if, for example, new drugs and therapies are to be developed. There is therefore a great tension between scientific accuracy on the one hand and medical-ethical necessity on the other. From this author’s point of view, Biological Statistical Mechanics avoids this dilemma by attaching only a *heuristic* value to the models of systems biology, without necessarily subscribing to systems ontology. Rather, this flexible and pragmatic attitude of the new project is compatible with different ontologies, so that the new approach invites a remarkable *ontological openness*. In addition, the great importance that the project’s founders attach to the indeterminacy and plasticity of biological entities enables them to enter into process ontological thinking, which is still terra incognita for the life sciences.

## 5. Conclusions

In the present article it is suggested that there are two inherently different philosophical methods of approaching the concept of organism: the *phenomenal analysis* and the *causal-ontological analysis of the organism*.

The term ‘phenomenal analysis of the organism’ summarizes approaches to the nature of the organism that are limited to the description of the phenomena essential for living beings. This article distinguishes between three successive levels of phenomenal analysis, in each of which the topic is treated on a higher level of abstraction: On the first and least abstract level, the term ‘organism’ refers to the body of a living being. As phenomenologically oriented philosophers have claimed, this approach implies an understanding of the essential differences between living and inanimate beings, which presupposes an intuitive, non-discursive approach to life that is anchored in our empathy with our own being alive. On the second level, a change of focus is proposed, which elevates the concept of the organism to a principle of identity. The term ‘organism’ means a nexus of reciprocally conditioning processes, through which (the nexus) maintains its own material form or varies it in a certain way, e.g., during embryogenesis, through the continuous exchange of its own material components. By focusing on the principle that regulates the continuous exchange of matter, this definition elevates *metabolism* to the most essential characteristic of the organism. Finally, on the third and most abstract level of the phenomenal analysis, emphasis is put on the *autonomy* of organismic self-organization: There is a rapidly growing body of publications in the fields of genetics, epigenetics and developmental plasticity that demonstrate the ability of cells and multicellular organisms to autonomously manipulate and reorganize their morphology, physiology, and genomes down to the molecular level of the latter. This reorganization enables organisms to radically restructure their bodies so that, even under extreme internal and external circumstances, they can create most of the conditions for the continuation and, if necessary, the targeted change of their metabolism. This ability is a distinctive characteristic of the organismic mode of being, which is also emphasized by the fact that the depth of organismic self-organization cannot even approximately be attained by the most complex examples of physical or chemical self-organization of inanimate matter.

The ‘causal-ontological analysis of the organism’ has accompanied biological thinking for millennia. It does not aim at describing the phenomenon of the organism, but at its theoretical justification or explanation. In ancient times and up to the first centuries of modern times, biological causality was viewed through the lens of substance ontology: the formation and maintenance of the organismic body was ascribed to an immaterial substance. In the 17th century, the ontology of substance had to cede the throne of scientific reason to systems ontology. The establishment of mathematical theoretical biology in the 1930’s and the rise of molecular biology twenty years later facilitated the breakthrough of systems-theoretically oriented materialistic interpretation of the organism. In the last twenty years, due to the emergence of synthetic and systems biology, organisms are considered complex dynamical systems of nonlinearly interacting biomolecules. Due to the commitment of systems biology to the practical service of the life sciences, in which the course of phenomena is described and predicted by different kinds of mechanisms, much of the systems biological research can be understood as the discovery, description, and creation of *mechanisms*. In biological modeling as well as in contemporary philosophy of science, ‘mechanisms’ are often represented by systems of differential equations. Systems biological mechanisms work successfully only if two conditions are met: First, in the equations there must be a strict separation between dynamic quantities, the so-called ‘variables’ that are calculated by the equations, and the static quantities, the so-called ‘parameters,’ which constrain the calculations. Second, the mathematical structure of the system of equations is invariable, which means that the results of the operations, i.e., the calculated values of the variables, have no influence on the operating mathematical structure. Based on these conditions, in this article, the factors that are causally relevant in the systems biological mechanisms were categorized using two differentiation criteria: *intrinsic* vs. *extrinsic* factors and *first-order* vs. *second-order* factors. Combinations of these categories make it possible to characterize the variables as ‘first-order intrinsic factors,’ the parameters as ‘first-order extrinsic factors,’ and the system of equations as a ‘second-order extrinsic factor.’ On the basis of these categories, a central result of the present article is the formulation of two rules that define the *logic of systems biological mechanisms*: (1) The number of first-order extrinsic factors exceeds several times the number of the first-order intrinsic factors and (2) the second-order factor of these mechanisms is always an extrinsic factor. Currently, new ontologies that are based on the hypothesis that the ultimate elements of physical reality are processes are being introduced into the theory of the organism and the philosophy of biology. One of the best-known versions of process ontology was introduced by Alfred North Whitehead in the 1920s and 1930s. Whitehead considers all elementary actual beings, of which the enduring micro-, meso-, and macroscopic entities of the physical universe consist, indivisible processes, which he calls ‘actual occasions.’ They are conceived as short-lived, indivisible (atomic) processes whose spatiotemporal expansion covers a broad spectrum, from quantum events to macroscopic conscious processes that occur in the brain. The conception of the ‘actual occasion’ is based on three metaphysical ideas: First, any ‘actual occasion’ is to a certain degree a creative being that strives to determine its own nature or essence. Aligned with its own self-determination, an ‘actual occasion’ is a teleologically acting, self-building being whose actualization unfolds due to increasingly emerging intrinsic tendencies. Second, ‘actual occasions’ are indissolubly related to a specific environment. Third, ‘actual occasions’ are conceived of as indissolubly mental-physical beings, which possess an elementary subjectivity. According to this ontology, living beings are understood as coherent groups of intertwined mental-physical processes that are teleologically coordinated elementary subjects. Whitehead introduced his understanding of process and living being against the background of all versions of mechanistic thought that were influential in the first half of the 20th century. It is no accident that if we apply the vocabulary of the logic of systems biological mechanisms to Whiteheadian process ontology, the logic of the ‘actual occasion’ appears as the exact opposite of the logic of mechanisms: As a creative being that determines its own essence through its self-formation, an ‘actual occasion’ is to be seen as a process whose inner causal structure is not pre-formed and fixed, but rather gradually emerges during this process of self-actualization. Accordingly, its teleological self-formation can only be controlled by a self-transforming causal structure or—formulated against the background of the categories of the logic of mechanisms—by a *second-order intrinsic factor*. Interestingly, the logic of the Whiteheadian process turns not only the second rule of mechanistic logic on its head, but also the first: During its emergence, an ‘actual occasion’ integrates environmental facts that initially exist extrinsically for the newly emerging process. But as the process of self-formation unfolds, it gradually processes these initially extrinsic facts, transforming them into intrinsic ones. When its self-determination is complete, the ‘actual occasion’ only contains causal factors, the nature of which was to a certain extent created by this ‘actual occasion’ itself or, in the words of the logic of mechanisms, it consists only of first-order intrinsic factors.

An essential thesis of this article is that a general process logic, which can provide the logical basis for a future theory of the organism, can be abstracted from Whitehead’s process ontology. The formulation of the *logic of the organism* is an important step in this direction. However, although this logic is compatible with Whiteheadian ontology, it was not derived from the latter. Rather, it summarizes in an abstract way new knowledge about the fundamental ability of organisms to do the following: Firstly, to subject the kinetic parameters of biochemical reactions to variations of many orders of magnitude, and secondly to reorganize their own physiological, morphological, and genetic structure. The essence of the logic of the organism is expressed by two rules: (1) In real organisms, the number of first-order intrinsic factors always exceeds by many times the number of first-order extrinsic factors, and (2) in real organisms, second-order factors are necessarily always intrinsic factors, i.e., there are no second-order extrinsic factors in real organisms. Obviously the contradiction between the logic of the organism and the logic of systems biological mechanisms could not be greater.

The logic of the organism is rooted in new insights into the essential ability of organisms to carry out radical rearrangements of their material structure autonomously. These findings, which emphasize the indeterminacy of living beings and their ontological distance from inorganic systems that have been investigated by physics, also constitute an important basis for the new project of Biological Statistical Mechanics. A main result of this article is the position that the only way out of the dilemma of the sharp contradiction between the logic of the organism and the logic of systems biological mechanisms is to use the latter mainly as a *heuristically* useful means of describing biological processes without ascribing an ontological value to these mechanisms. In this author’s opinion, this suggestion is already at least implicitly followed by the proponents of Biological Statistical Mechanics, as they are aware of the fact that organisms are much more dynamic than they are described in the current models. The new project allows for a spectrum of organismic ontologies that is considerably wider than the implicit ontology of contemporary theoretical and systems biology. This is one of the reasons why this author thinks that the new project of Biological Statistical Mechanics can be supported by the process ontological understanding of the organism. *Just as many roads lead to Rome, so do many lead to the process thought*—*one of the most rewarding of them probably leads through the gate of the logic of the organism.*

## Data Availability

The data is contained within the article.
